# Can we Save the rectum by watchful waiting or TransAnal surgery following (chemo)Radiotherapy versus Total mesorectal excision for early REctal Cancer (STAR‐TREC)? Protocol for the international, multicentre, rolling phase II/III partially randomized patient preference trial evaluating long‐course concurrent chemoradiotherapy versus short‐course radiotherapy organ preservation approaches

**DOI:** 10.1111/codi.16056

**Published:** 2022-03-24

**Authors:** Simon P. Bach, the STAR‐TREC Collaborative

**Affiliations:** ^1^ D3B [Drugs, Devices, Diagnostics and Biomarkers] Cancer Research UK Clinical Trials Unit Birmingham UK

**Keywords:** chemoradiotherapy, circulating free tumour DNA, complete response, early rectal cancer, organ preservation, short‐course radiotherapy, transanal endoscopic microsurgery, watch and wait

## Abstract

**Aim:**

Organ‐saving treatment for early‐stage rectal cancer can reduce patient‐reported side effects compared to standard total mesorectal excision (TME) and preserve quality of life. An optimal strategy for achieving organ preservation and longer‐term oncological outcomes are unknown; thus there is a need for high quality trials.

**Method:**

Can we Save the rectum by watchful waiting or TransAnal surgery following (chemo)Radiotherapy versus Total mesorectal excision for early REctal Cancer (STAR‐TREC) is an international three‐arm multicentre, partially randomized controlled trial incorporating an external pilot. In phase III, patients with cT1‐3b N0 tumours, ≤40 mm in diameter, who prefer organ preservation are randomized 1:1 between mesorectal long‐course chemoradiation versus mesorectal short‐course radiotherapy, with selective transanal microsurgery. Patients preferring radical surgery receive TME. STAR‐TREC aims to recruit 380 patients to organ preservation and 120 to TME surgery. The primary outcome is the rate of organ preservation at 30 months. Secondary clinician‐reported outcomes include acute treatment‐related toxicity, rate of non‐operative management, non‐regrowth pelvic tumour control at 36 months, non‐regrowth disease‐free survival at 36 months and overall survival at 60 months, and patient‐reported toxicity, health‐related quality of life at baseline, 12 and 24 months. Exploratory biomarker research uses circulating tumour DNA to predict response and relapse.

**Discussion:**

STAR‐TREC will prospectively evaluate contrasting therapeutic strategies and implement new measures including a smaller mesorectal target volume, two‐step response assessment and non‐operative management for complete response. The trial will yield important information to guide routine management of patients with early‐stage rectal cancer.

## BACKGROUND

Bowel cancer is the third most common tumour with over 42 000 new UK cases annually, 447 000 across Europe and 1.93 million worldwide, of which one‐third are located in the rectum [1]. Historically, rectal tumours presented symptomatically at a relatively advanced stage; however, the introduction of bowel screening allied with improved access to diagnostic testing has increased the proportion of patients diagnosed with early‐stage rectal cancer. Currently 25% of total mesorectal excision (TME) operations performed in the UK are for non‐irradiated T1 and T2N0 tumours [2].

Surgery alone to remove the rectum, adhering to the principles of TME, is the current standard of care for treatment of early‐stage rectal cancer [3, 4. Unfortunately, rectal resection with TME is associated with substantial morbidity, occasional mortality and considerable impact upon patients' health‐related quality of life (HRQoL). Patient groups and the public recognize the potential benefits of an organ preservation approach for the treatment of early‐stage rectal cancer to reduce the morbidity associated with radical surgery and consider this a top research priority [5].

The feasibility of an organ‐sparing approach for patients with locally advanced rectal cancer has been demonstrated by non‐operative management or ‘watch and wait’, following complete clinical response (cCR) to preoperative chemoradiotherapy (CRT) [6]. Evaluation of organ preservation strategies for early‐stage rectal cancer has also gathered momentum in recent years. Initial studies focused upon optimized platforms for transanal local excision such as transanal endoscopic microsurgery (TEM). However, this approach does not achieve acceptable rates of local disease control for the majority of patients with proven rectal malignancy [7]. In addition, the risk of local recurrence following transanal excision is associated with the presence of specific high‐risk histopathological features that are only evaluable, and thereby appreciated, once the tumour is removed [8]. Several studies including CARTS, ACOSOG Z6041 and GRECAR 2 have evaluated conventional CRT combined with local, transanal excision but, despite encouraging long‐term outcomes with organ preservation rates of 64%–91% and isolated local relapses of 5% or less [9, 10, 11, initial reports highlighted cumulative toxicities from multiple treatments that negated the benefits of an organ‐preserving approach [12, 13, 14. Systematic reviews currently point to a lack of high quality evidence as a barrier to adoption of organ preservation for patients with early rectal cancer who are considered suitable for TME surgery [15, 16.

While CRT is commonly used for organ preservation strategies, short‐course radiotherapy (SCRT) may also be considered. The efficacy of preoperative SCRT compared with CRT for prevention of local relapse is supported by two phase III trials in patients with radically excised rectal cancer, where SCRT showed benefits in terms of reduced toxicity [17, 18. SCRT with delayed TEM provided safe and effective organ preservation for frail, elderly patients with cT1 and T2N0 rectal cancer [19]. The TREC trial randomized patients with cT1 and T2N0 rectal cancer to organ preservation via SCRT and TEM versus TME surgery [20]. Organ preservation was associated with fewer serious complications, reduced acute patient‐reported toxicity, and consequently had little impact on HRQoL and function at 3 months compared to TME surgery. Sustained benefits in overall HRQoL, social function, body image, and decreased embarrassment about bowel function were also observed up to 3 years with organ preservation versus TME surgery. Organ preservation was achieved in 70% of cases and the risk of unsalvageable local recurrence was extremely low.

TREC [20] and CARTS [13] provided the basis for the design of the international STAR‐TREC study to refine and further evaluate novel organ‐preserving strategies for early rectal cancer utilizing radiotherapy and local transanal microsurgery. Key aims were to increase the effectiveness of organ‐preserving treatment while simultaneously reducing treatment‐related toxicity [21]. STAR‐TREC introduced a smaller mesorectal (only) target volume, risk adapted for early‐stage tumours [22, 23, a standardized response assessment up to 20 weeks after commencement of CRT, and non‐operative management of complete response. An external pilot (phase II) demonstrated that it was possible to randomize and accelerate recruitment to a three‐way randomized design [24]. STAR‐TREC transitioned to a full phase III trial in 2020 via a major protocol amendment (version 4.0, 10 October 2019), introducing a modified study design that allows patients to select either radical surgery or organ preservation. This was based upon feedback from patients who enrolled in phase II, extensive patient and public consultation endorsing patient choice [5], and emergence of more mature trial data highlighting the relative safety of an organ‐saving approach compared to TME surgery [20]. Key patient priorities are achieving organ preservation, maintaining quality of life, and provision of safe treatment including the ability to salvage any local recurrence [5]. The objective of STAR‐TREC is to evaluate two contrasting organ preservation strategies, using either long‐course CRT or SCRT for the treatment of early‐stage rectal cancer, in terms of organ preservation rates, toxicity (clinician‐ and patient‐reported) and HRQoL. Consequently, in STAR‐TREC the leading question relates to the overall success of an organ preservation strategy, followed by which organ preservation arm facilitates greater organ preservation with the lowest likelihood of toxicity. The study will also establish the oncological safety of this approach, where unsalvageable pelvic failure is a rare event. STAR‐TREC follows the recent international consensus statement for the reporting of key outcome measures of organ preservation [25].

## METHOD

### Study design

This is an international multicentre, rolling phase II/III trial. Phase II incorporated three‐way randomization (1:1:1) between (i) TME surgery, (ii) organ preservation via mesorectal SCRT or (iii) organ preservation via mesorectal CRT [24]. The modified phase III design is a partially randomized, patient preference study, where patients select either (a) TME surgery or (b) organ preservation. Those who prefer organ preservation are randomized (1:1) between (i) organ preservation via mesorectal SCRT or (2) organ preservation via mesorectal CRT.

#### External phase II

An external phase II study was conducted over 2 years, following a 6‐month initial setup, to assess the feasibility of a large, multicentre randomized trial comparing radical surgery versus organ‐saving treatment and selective transanal microsurgery [24]. The primary end‐point of phase II was the recruitment rate at 12 and 24 months with targets of ≥4 and ≥6 patients randomized per month respectively for a total accrual of 120 international cases. The core secondary end‐points in phase II were (i) procurement of funding by one international partner, (ii) trial opening by one international partner and (iii) an organ‐saving rate >50% at 12 months (following randomization) in the experimental arms. The independent Data Monitoring Committee (DMC) deemed that these criteria were satisfied and transition to phase III was via separate funding applications and a prespecified protocol amendment. Data collected from the phase II component will also be analysed in accordance with the phase III outcome measures.

### Participants, interventions and outcomes

#### Study setting

This is an international hospital‐based study in the UK, Netherlands and Denmark (and planned to open in Belgium and Sweden). Potential candidates are identified in the endoscopy suite following (i) investigation of new bowel symptoms, (ii) personal bowel surveillance or (iii) bowel screening and are referred to a colorectal surgeon or the colorectal cancer multidisciplinary team (MDT), where eligibility is confirmed following review of clinical data (Table1).

**TABLE 1:** STAR‐TREC trial registration data

Data category	Information
Primary registry and trial identifying number	ISRCTN14240288
Date of registration in primary registry	20 October 2016
Secondary identifying numbers	EudraCT 2016‐000862‐49 ClinicalTrials.gov: NCT02945566
Source(s) of monetary or material support	Cancer Research UK, Dutch Cancer Society, Danish Cancer Society, Against Cancer Flanders
Primary sponsor	University of Birmingham, Birmingham, B15 2TT, UK Email: researchgovernance@contacts.bham.ac.uk
Secondary sponsor(s)	
Contact for public queries	STAR-TREC@Trials.bham.ac.uk
Contact for scientific queries	STAR-TREC@Trials.bham.ac.uk
Public title	Can we Save the rectum by watchful waiting or TransAnal surgery following (chemo)Radiotherapy versus Total mesorectal excision for early REctal Cancer
Scientific title	Can we Save the rectum by watchful waiting or TransAnal surgery following (chemo)Radiotherapy versus Total mesorectal excision for early REctal Cancer
Countries of recruitment	Current: UK, Netherlands, Denmark Future: Belgium, Sweden
Health condition(s) or problem(s) studied	Early rectal cancer
Intervention(s)	Randomized comparator: organ preservation with short‐course radiotherapy A total dose of 25 Gy in five daily fractions over a total time of 1 week, using 5 Gy per fraction
	Randomized comparator: organ preservation with long‐course chemoradiotherapy A total dose of 50 Gy in 25 daily fractions over a total time of 5 weeks, using 2.0 Gy per fraction, combined with capecitabine 825 mg/m^2^ twice daily on radiotherapy days
	Non‐randomized comparator: radical total mesorectal excision Encompassing reconstructive (low anterior resection) and non‐reconstructive (abdominoperineal excision, low Hartmann's procedure) approaches
Key inclusion and exclusion criteria	Ages eligible for study: ≥16 years in UK, ≥18 years in other countries Sexes eligible for study: both Accepts healthy volunteers: no
	Main inclusion criteria: Biopsy proven adenocarcinoma of the rectum MRI or ERUS staged TX/T1‐3b, NX/N0, MX/M0 rectal tumour The MDT determines that the following treatment options are all reasonable and feasible: (a) TME surgery, (b) CRT, (c) SCRT and (d) TEM ECOG performance status 0–1 Willing and able to consent
	Main exclusion criteria: Concomitant or previous malignancies within 3 years prior to trial entry, except those that in the opinion of the MDT are unlikely to relapse within 3 years or lead to death within 5 years MRI node positive (≥N1, defined by protocol guidelines) MRI extramural vascular invasion (mriEMVI) present (defined by protocol guidelines) MRI defined mucinous tumour Mesorectal fascia threatened by tumour (≤1 mm on MRI or ERUS) Maximum tumour diameter >40 mm (measured from everted edges on either sagittal MRI or ERUS examination) Anterior tumour location above the peritoneal reflection on MRI or ERUS No residual luminal tumour following endoscopic mucosal resection Prior pelvic radiotherapy Definite evidence of regional or distant metastases (M1) in the opinion of the MDT Uncontrolled cardiorespiratory comorbidity (inadequately controlled angina or myocardial infarction or arrhythmia within 6 months prior to trial entry) Known complete dihydropyrimidine dehydrogenase deficiency Known Gilbert's disease Taking coumarin‐derivative oral anticoagulants that cannot be stopped or substituted by low molecular weight heparin Taking metronidazole, phenytoin, sorivudine or its analogues, such as brivudine Women who are pregnant or lactating
Study type	Interventional
	Open, parallel assignment, partially randomized intervention model Patients will choose organ preservation or standard surgery. Those who prefer organ preservation will be randomized 1:1 between (i) organ preservation with mesorectal CRT versus (ii) organ preservation with mesorectal SCRT. Those who prefer standard surgery or have no preference will undergo standard TME surgery without neoadjuvant radiotherapy treatment
	Primary purpose: treatment
	Rolling phase II–III
Date of first enrolment	14 June 2017
Target sample size	380 patients randomized to the organ preservation arms (CRT and SCRT) Estimated 120 patients recruited to the standard surgery comparator arm
Recruitment status	Recruiting
Primary outcome(s)	See Table 3
Key secondary outcomes	See Table 3

Abbreviations: CRT, chemoradiotherapy; ECOG, Eastern Cooperative Oncology Group; ERUS, endorectal ultrasound; MDT, multidisciplinary team; SCRT, short‐course radiotherapy; TEM, transanal endoscopic microsurgery; TME, total mesorectal excision.

#### Eligibility criteria

All patients must have biopsy proven adenocarcinoma of the rectum, staged as ≤mrT3b, that is, ≤5 mm of mesorectal invasion, Eastern Cooperative Oncology Group (ECOG) performance status 0–1, and the MDT must consider that both TME surgery and organ‐saving therapy are both reasonable and feasible treatments. Major exclusions include primary tumours >40 mm in maximum diameter (sagittal MRI or endorectal ultrasound [ERUS]), mr ≥ N1, and mesorectal fascia threatened by tumour (≤1 mm on MRI). A comprehensive eligibility checklist is provided in Table 2.

**TABLE 2:** Inclusion and exclusion criteria for the phase III STAR‐TREC study

Inclusion criteria	Exclusions
**All patients**	**All patients**
1. Biopsy proven rectal adenocarcinoma	1. Previous malignancy <3 years (patients may be included where relapse within 3 years or death within 5 years is deemed unlikely)^b^
2. ≤mrT3b (≤5 mm of mesorectal invasion)	2. Unequivocal metastatic disease staged as M1
3. ECOG 0–1	3. mr ≥ N1^c^
4. MDT considers TME, CRT, SCRT, TEM are all reasonable and feasible	4. mriEMVI positive^c^
5. Willing and able to provide informed consent	5. MRI defined mucinous tumour
	6. Mesorectal fascia threatened by tumour (≤1 mm on MRI)
**Only organ preservation: female of childbearing potential**	7. Maximum tumour diameter > 40 mm (MRI or ERUS)
1. Negative pregnancy test within 7 days of study entry	8. Tumour situated anteriorly above peritoneal reflection (MRI or ERUS)
2. Agree to use medically approved contraception^a^	9. No residual macroscopic tumour following EMR
	10. Contraindications to CRT^d^
**Only organ preservation: non‐sterilized male with a partner of childbearing potential**	11. Age 16 years (UK), <18 years (other countries)
1. Agree to use medically approved contraception^a^	

Abbreviations: CRT, chemoradiotherapy; ECOG, Eastern Cooperative Oncology Group; EMR, endoscopic mucosal resection; ERUS, endorectal ultrasound; MDT, multidisciplinary team; mriEMVI, MRI extramural vascular invasion; SCRT, short‐course radiotherapy; TEM, transanal endoscopic microsurgery; TME, total mesorectal excision.

^a^ From trial entry until 6 months after the end of study treatment.

^b^ In the opinion of the MDT.

^c^ Defined by protocol guidelines.

^d^ Previous pelvic radiotherapy, uncontrolled cardiorespiratory comorbidity, known complete dihydropyrimidine dehydrogenase deficiency, known Gilbert's disease (hyperbilirubinaemia), taking coumarin‐derivative anticoagulants (e.g., warfarin) that cannot be discontinued at least 7 days prior to starting treatment or substituted by low molecular weight heparin, taking phenytoin or sorivudine or its chemically related analogues such as brivudine within 4 weeks of trial entry, taking metronidazole at study entry, pregnant or lactating women, history of severe and unexpected reactions to fluoropyrimidine therapy.

#### Recruitment

STAR‐TREC is a hospital‐based study. Candidates will generally be identified in the endoscopy suite following referral (i) for the investigation of new bowel symptoms, (ii) as part of a personal bowel surveillance programme or (iii) through national bowel screening. Subjects will then be referred to either a colorectal surgeon or the colorectal cancer MDT meeting. Recruiting trial sites are likely to act as ‘regional hubs’ for the early rectal cancer service. They will specialize in organ‐saving techniques while being able to provide standard (radical surgery) treatment. Following informed consent, which will be conducted by medically qualified individuals in accordance with good clinical practice standards, confirmation of eligibility and trial entry will be conducted using electronic remote data capture.

#### Surgical interventions

Standard primary TME surgery may encompass both reconstructive and non‐reconstructive approaches to rectal resection adhering to the principles of TME surgery. The former includes low anterior resection, the latter abdominoperineal excision or low Hartmann's procedure. Surgeons may employ either minimally invasive, open or hybrid approaches to TME surgery. The quality of surgery will be measured using a standardized histopathological assessment that grades whether surgery was performed according to the principles of TME.

Local transanal excision is performed either by TEM or an equivalent single‐port transanal technique, to remove the tumour site and underlying muscularis propria en bloc, aiming for a 10 mm margin of normal mucosal tissue. A thin layer of mesorectal fat is also removed; however, the mesorectum is not extensively dissected. The specimen is pinned out in the operating room prior to fixation to facilitate accurate evaluation of margin status. It is recommended that the defect is sutured closed to promote healing.

Patients who undergo planned conversion from transanal excision to secondary TME, based upon the presence of designated high‐risk histopathological features in the local excision specimen, are expected to have surgery 8–16 weeks after TEM, allowing time for post‐surgical inflammation to settle.

#### (Chemo)radiotherapy interventions

Long‐course CRT consists of a total dose of 50 Gy in 25 fractions, combined with capecitabine administered at a dose of 825 mg/m^2^ twice per day on radiotherapy days only. The protocol includes general guidelines for management of common toxicities related to capecitabine and dose reduction rules based on the relevant product information. SCRT consists of a total dose of 25 Gy in five fractions of 5 Gy, preferably on five consecutive days. Radiotherapy for organ preservation in this group of early cancers is primarily aimed at tumour downstaging and can therefore be restricted to the peritumoural area including the primary tumour and the mesorectum. The treated mesorectal volume now corresponds to the volume that would be resected in a TME resection. Elective irradiation of lateral or presacral lymph nodes cranial to or above the mesorectal volume is thus not indicated. This results in a significant reduction of the irradiated target volume and is expected to result in less toxicity [22, 23. Detailed radiotherapy guidelines and an international quality assurance programme are employed to ensure high quality radiotherapy delivery. Timing of response assessment is calculated from the start of radiotherapy treatment, rather than on completion, to improve comparison of tumour regression between SCRT and CRT.

#### Primary outcome

The primary outcome is the proportion of patients with successful organ preservation at 30 months from the start date of CRT. Organ preservation is considered to have failed if (i) the rectum is removed, (ii) the patient develops unequivocal locoregional cancer recurrence or (iii) the patient has a stoma. Analysis of the phase III outcomes will include all the relevant patients recruited during both the phase II and phase III components of the trial (Table 3).

**TABLE 3:** Summary of primary and secondary end‐points for the STAR‐TREC study (phase III)

Randomized comparison between organ‐preserving strategies	Analysis incorporating standard surgery comparator
Primary end‐point	
Proportion of patients with successful organ preservation at 30 months from the start date of (chemo)radiotherapy^a^	No
Patient‐reported secondary outcomes^b^	
Symptomatic toxicity	Yes
Health‐related quality of life	Yes
Health economics	Yes
Treatment decision regret^c^	Yes
Clinician‐reported secondary outcomes	
Acute treatment‐related toxicity up to 30 days following completion of (chemo)radiotherapy or primary surgery	Yes
Proportion of patients with complete response^d^	No
Proportion of patients who have transanal local excision	No
Time to event of organ loss; length of time from the start date of trial treatment until TME surgery	No
Non‐regrowth pelvic tumour control to 36 months^e^	Yes
Metastasis‐free survival to 36 months^f^	Yes
Non‐regrowth disease‐free survival to 36 months^g^	Yes
Overall survival to 60 months^h^	Yes
Exploratory cftDNA biomarker studies	
Retrospective sensitivity and specificity analysis to evaluate CR	No
Retrospective sensitivity and specificity analysis to evaluate relapse	No

Abbreviations: cftDNA, circulating free tumour DNA; CR, complete response; TME, total mesorectal excision.

^a^ Organ preservation is considered to have failed if (i) the rectum is removed, (ii) the patient develops unequivocal locoregional cancer recurrence or (iii) the patient has a stoma.

^b^ Measured using European Organization for Research and Treatment of Cancer (EORTC) Quality of Life Questionnaire (QLQ) CR29 and C30, EuroQol EQ‐5D, Low Anterior Resection Syndrome score and International Consultation on Incontinence Questionnaire Male Lower Urinary Tract Symptoms Module (ICIQ‐MLUTS)/International Consultation on Incontinence Questionnaire Female Lower Urinary Tract Symptoms Module (ICIQ‐FLUTS) at 12 and 24 months compared to baseline.

^c^ Measured using the validated decision regret scale questionnaire at 24 months.

^d^ Complete response is defined by the presence of all the following criteria: (i) satisfactorily passed first (11–13 week) clinical assessment with no evidence of tumour progression, (ii) endoscopy at 16–20 weeks shows no evidence of mucosal tumour, mucosal ulceration or submucosal swelling but only a flat, white scar remains ± telangiectasia, and (iii) there is no palpable tumour upon digital rectal examination.

^e^ Defined as the length of time from the start date of trial treatment or date of initial surgery until death (any cause) or development of unequivocal pelvic recurrence but not including patients who preferred organ preservation and developed local regrowth which was resected with clear margins using standard TME surgery.

^f^ Defined as the length of time from the start date of trial treatment or date of initial surgery until death (any cause) or detection of distant metastasis.

^g^ Defined as the length of time from the start date of trial treatment or date of initial surgery until death (any cause), detection of local pelvic recurrence or distant metastasis but not including patients who developed local regrowth which was resected with clear margins using standard TME surgery.

^h^ Defined as the length of time from the start date of trial treatment or date of initial surgery until death (any cause).

#### Secondary outcomes

Major patient‐ and clinician‐reported secondary outcome measures are presented in Table 3. Quality of life and HRQoL are measured using European Organization for Research and Treatment of Cancer (EORTC) Quality of Life Questionnaire (QLQ) CR29 and C30, EuroQol EQ‐5D, Low Anterior Resection Syndrome (LARS) score and International Consultation on Incontinence Questionnaire Male Lower Urinary Tract Symptoms Module (ICIQ‐MLUTS)/International Consultation on Incontinence Questionnaire Female Lower Urinary Tract Symptoms Module (ICIQ‐FLUTS) at 12 and 24 months compared to baseline.

#### Participant timeline

**FIGURE 1:**
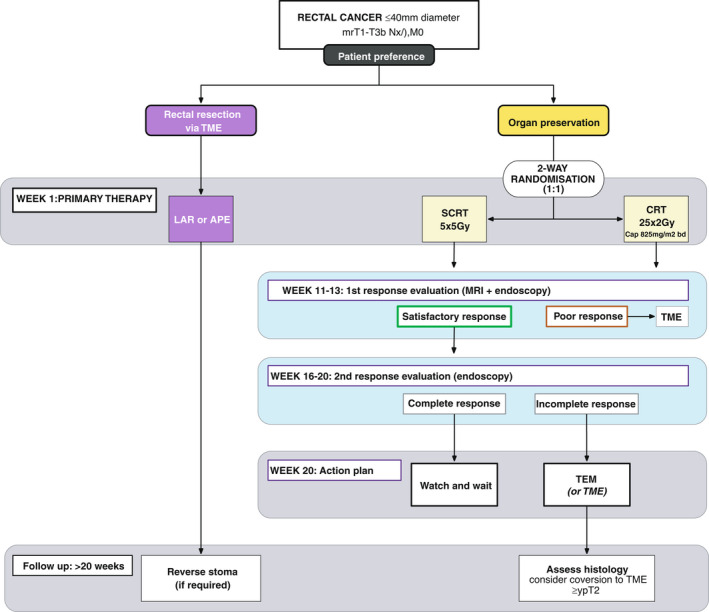
Flowchart of the inclusion, randomization and management of the study subjects in the STAR‐TREC phase III trial. APE, abdominoperineal excision; CRT, chemoradiotherapy; LAR, low anterior resection; SCRT, short‐course radiation therapy; TEM, transanal endoscopic microsurgery; TME, total mesorectal excision

In the modified phase III design patients can select either (a) TME surgery or (b) organ preservation. Those who prefer organ preservation are randomized (1:1) between (i) organ preservation via mesorectal SCRT or (ii) organ preservation via mesorectal CRT (Figure 1). In the two organ‐saving arms, response assessment will take place at 11–13 weeks from the start of CRT and again at 16–20 weeks from the start; this determines the next treatment step. Initial composite assessment at 11–13 weeks using MRI and endoscopy identifies a small proportion of cases where radiotherapy has had little impact on tumour dimensions so patients can convert to TME surgery. Individuals whose tumours demonstrate a satisfactory response at this time point will be examined once again at 16–20 weeks by endoscopy to determine if they achieved complete clinical response (cCR). The protocol defines cCR as a flat white scar, with no evidence of mucosal tumour, mucosal ulceration or submucosal swelling (see Appendices [Link], [Link], [Link], [Link]). It is anticipated that this interval between assessments will allow for additional tumour regression and resolution of acute radiotherapy reactions, facilitating more precise diagnosis of cCR. Active surveillance will be performed in the case of a cCR, while patients with partial response will usually progress to local transanal excision unless the clinical team believe that TME is more appropriate. All patients are expected to be assigned to one of the three treatment groups by week 20: (a) non‐operative management; (b) local transanal excision; or (c) conversion to TME surgery. Histopathological assessment will identify high‐risk features in TEM specimens following SCRT or CRT that predict future local failure. These are margin involved by adenocarcinoma (R1 ≤1 mm), primary tumour stage ≥ypT2 or ypN+ (occasional lymph nodes are retrieved). Patients who exhibit one high‐risk feature are strongly recommended to consider conversion to TME, while two or more high‐risk features prompt a very strong recommendation.

#### Follow‐up schedules

Patients who select organ preservation commit to regular endoscopic and MRI examination of the rectum, quarterly in the first year and at least 6 monthly thereafter to 3 years. Full surveillance details for both organ preservation and TME surgery are provided in Table 4.

**TABLE 4:** Surveillance schedule for STAR‐TREC phase III

	6 months	9 months	12 months	18 months	24 months	30 months	36 months
Organ preservation							
Physical examination	x	x	x	x	x	x	x
Colonoscopy		x^a^					
Rectal endoscopy	x	x	x	x	x	x	x
CT thorax, abdomen, pelvis (TAP)^c^					x		x
MRI rectum	x	x	x	x	x	x	x
Standard TME surgery							
Physical examination	x		x		x	x	x
Colonoscopy		x^a^					
CT (TAP)					x		x
MRI rectum					X^b^		x^b^

Abbreviations: CT, computed tomography; TME, total mesorectal excision.

^a^ At least one colonoscopy to be performed in the first 3 years after treatment in accordance with national guidance.

^b^ Only required if CT pelvis is not performed.

^c^ CT pelvis optional if MRI pelvis performed at 24 and 36 months.

#### Sample size

In a comparison of two active treatments, the goal of the analysis is to identify whether either is superior. As the difference between two active treatments may be relatively small, conventional statistical significance (*P* < 0.05) is not an appropriate criterion because important differences may not be statistically significant [26]. Using only significant differences to establish the superiority of an intervention also increases the risk of substantially exaggerating the treatment effect as significance will only occur where, by chance, the estimated treatment effect is large. Instead, we will use Bayesian methods to estimate the probability that each treatment is superior, as well as providing estimates of the treatment effect and its uncertainty. There is no fixed threshold for regarding a treatment as sufficiently likely to be superior that it should be adopted in preference to the alternative, because this is a clinical judgement that will depend on other effects of the intervention and patient characteristics. We have set the timescale for recruitment to the phase III study at 4 years, which is acceptable to the clinical community, and the organ preservation sample size achievable within this is expected to be 380 (including 80 patients randomized to organ‐preserving treatments from phase II). The expected incidence of the primary outcome is approximately 60%, and we predict that a 10% difference between treatment arms will be regarded as clinically important, while smaller differences are unlikely to promote therapy change due to the presence of established geo‐regional practices relating to the use of CRT versus SCRT. A sample size of 380 allowing for an 8% dropout yields 350 evaluable cases, which our simulations have shown would have a probability of around 80%, or higher, of correctly identifying the superior treatment, irrespective of whichever is better, if the true difference is 10%. If the organ preservation rate turns out to be higher than anticipated (70% or 80%), the probability of detecting a 10% improvement remains over 85%. In all cases, the probability of identifying the wrong treatment as superior is very low (less than 1%). It is estimated that an additional 120 TME cases will be recruited to the partially randomized TME reference group, comprising 40 cases randomized to TME in phase II and a further 80 non‐randomized cases recruited in phase III.

#### Translational substudy

Biomarker research is exploratory and will investigate the potential future role of circulating free tumour DNA (cftDNA) associated with the persistence of tumour tissue to direct or support decisions to escalate medical or surgical therapy. Six sets of blood samples are collected from organ preservation patients explicitly consenting to this optional substudy at baseline, second response assessment, completion of treatment (6 months) and during follow‐up (12, 18, 24 months) using vials shipped without processing. Retrospective sensitivity and specificity analysis will evaluate the utility of cftDNA measurement for initial response assessment, that is, partial versus complete response, and also for patient follow‐up, to facilitate early identification of cancer regrowth or relapse. Primary tumour tissue taken before and after radiotherapy will also be collected.

### Assignment of interventions

Randomization will be achieved by a remote central web‐based service based at the Cancer Research (UK) Clinical Trials Unit (CRCTU), Birmingham. Randomization will use a minimization procedure with the following variables: mriT stage (≤T3a and T3b) and country of recruitment. STAR‐TREC is an open‐label study and neither patients nor site teams are masked to treatment allocation. To avoid any possibility of the treatment allocation becoming too predictable, a random factor was included within the algorithm whereby for a proportion of the allocations true randomization is implemented.

### Data collection, management and analysis

Data collection is carried out using patient notes, case report forms and validated quality of life questionnaires (EORTC QLQ‐C30, EORTC QLQ‐CR29, EuroQoL EQ‐5D‐3L, ICIQ‐MLUTS, ICIQ‐FLUTS, LARS score, decision regret scale). From June 2021 all data except patient questionnaires are entered onto an electronic case report form (by a member of the site staff). Regular data quality checks will be performed as per the Quality Management Plan. All data are handled in accordance with the Data Protection Act and General Data Protection Regulation. Data backups are stored in secure fireproof locations and test restorations are performed on a regular basis. After completion of the trial all essential trial documentation and source documents (e.g., signed informed consent forms, investigator site files, pharmacy file, participants' hospital notes, copies of case report forms etc.) are securely retained for at least 25 years.

#### Statistical methods

Full details regarding the statistical methods are given in the trials statistical analysis plan (SAP) version 1.0 dated 7 October 2021, authored by V. Homer, S. Gates, S. Bach and L. Navarro‐Nuñez, available on request. An overview is given here. The analytical strategy aims to evaluate whether (a) either organ‐saving strategy is superior in terms of achieving organ preservation, requirement for further surgery, treatment‐related toxicity, HRQoL and other outcomes; (b) either or both organ‐saving strategies lead to an acceptable rate of organ preservation of at least 50%. The primary analyses in STAR‐TREC phase III will be conducted according to the intention to treat principle, where participants are analysed in the treatment group to which they were randomized, regardless of the treatment received.

A key advantage of using a Bayesian statistical approach is that it quantifies the probability of benefit, or the probability of benefit of at least a given size, for each treatment arm. Hence, even if differences were relatively small, and conventional frequentist analysis with statistical significance (*P* < 0.05) would be inappropriate, the analysis can provide information that can inform clinical decisions directly about which treatment should be preferred based upon rates of non‐operative management, toxicity profiles and HRQoL.

The primary analysis will be the randomized comparison of the two organ‐preserving treatments using Bayesian logistic regression models, with adjustment for baseline covariates known to be related to outcomes (including severity of disease, age, sex and country of recruitment). Other dichotomized outcomes will be analysed similarly. HRQoL (and continuous outcomes) will be analysed using hierarchical repeated measures models to model each patient's trajectory through time, to account for the multiple data points per patient. Analysis of toxicity will evaluate the rate of major treatment‐related complications in each group.

Secondary analyses will incorporate the partially randomized standard surgery comparator. This partially randomized TME surgery reference group will provide data relating to the incidence of major treatment‐related toxicity and HRQoL following TME surgery for early‐stage rectal cancer, without the use of radiotherapy. Although the non‐randomized analysis means that conclusions must be treated with extra caution, this analysis will provide valuable additional information for patients with early‐stage rectal cancer who are balancing the risks and benefits of different treatment strategies.

In addition to the aforementioned, we will analyse the overall outcome for each patient using a patient‐centred ranked composite outcome analysis which will classify each patient's overall outcome, based on mortality, organ preservation, treatment‐related toxicity, need for surgery and quality of life, into an ordinal scale. The ranking of outcome categories will be determined by consensus among the investigators during the conduct of the trial and will be reviewed (and potentially modified) by a sample of clinicians and patients. The ordinal overall outcome measure will be used to compare the randomized organ preservation groups, using ordinal regression models. The main advantages of this approach are that it is more relevant to patients because it considers patients' overall outcome, and it enables all patients’ outcomes to contribute to the analysis [27, 28. This analysis will also identify to what extent patients who do not achieve organ preservation may be disadvantaged by their treatment choice compared to those who select TME surgery.

### Monitoring

The University of Birmingham is the coordinating sponsor for this international study and is also the National Coordinating Centre (NCC) for the UK. The coordinating sponsor has in force a Public Liability Policy and Clinical Trials Policy which provide cover for claims for negligent harm arising from the design or management of the research. Each country will appoint a National Coordinating Investigator and an NCC who will take national responsibility for the study and manage the trial in accordance with the trial protocol, and their standard policies and procedures. Central and site monitoring activities to ensure compliance with the protocol and applicable regulations will be conducted using a risk‐based approach as documented in the trial risk assessment and quality management plan. The trial management group is composed of representatives from each NCC and the trial team at CRCTU (Table 5). They are responsible for day to day running of the trial. The collection and reporting of adverse events are in accordance with the Medicines for Human Use Clinical Trials Regulations 2004 and its subsequent amendments. Events are graded using the National Cancer Institute's Common Terminology Criteria for Adverse Events version 4.03 and also the Clavien–Dindo classification of postoperative complications [29, 30. The reporting period is from the date of commencement of the protocol‐defined treatment until 30 days after the administration of the last trial treatment. The independent DMC is provided with safety data for each treatment arm, including frequency of adverse events and serious adverse events for all three arms. In conjunction with the Trials Steering Committee (TSC), the DMC will advise on the continuation or early stoppage of the trial in the unlikely event that there are concerns over harm to participants.

**TABLE 5:** Charter, responsibilities and membership of the STAR‐TREC trial monitoring committees

Data Monitoring Committee (DMC)	
Responsibilities	The aims of the DMC are (i) to protect and serve STAR‐TREC trial patients (especially in relation to safety) and to assist and advise the Sponsor, via the Chief Investigator and other members of the Trial Management Group (TMG), so as to protect the validity and credibility of the trial; and (ii) to safeguard the interests of participants, assess the safety and efficacy of the interventions, and monitor the overall conduct of the clinical trial A copy of the DMC Charter is available upon request
Relationships	During the recruitment period, interim analyses of the trial's progress including updated figures on recruitment, data quality and completeness, main outcomes and safety will be supplied in strict confidence to the DMC The DMC is independent of the study organizers and advisory to the Sponsor, who remains legally responsible for the conduct of the trial. The DMC should make comments, requests and recommendations to the Chair of the Trials Steering Committee (TSC) for consideration. The Trial Statistician should be kept informed (in strictest confidence) of any discussions to ensure that appropriate records can be maintained on behalf of the Sponsor within the Trial Master File
Membership	Professor Alex Mirnezami, Professor of Surgical Oncology at University Hospital Southampton Foundation Trust, Chair and Surgical Lead Dr Louise Hiller, Associate Professor at Warwick Clinical Trials Unit, University of Warwick, Statistical Lead Dr Amandeep Singh Dhadda, Consultant Clinical Oncologist at Hull University Teaching Hospitals NHS Trust, Radiation Oncology Lead
Trial Steering Committee (TSC)	
Responsibilities	The aims of the TSC are (i) to provide independent supervision and oversight of the trial on behalf of the Sponsor and the Trial Funder so as to protect trial patients and the validity and credibility of the trial; (ii) to assist and provide expert advice to the Chief Investigator and other members of the TMG; and (iii) to take the ultimate decision for the continuation of the trial A copy of the TSC Charter is available upon request.
Relationships	During the recruitment period, the TSC will monitor and supervise the progress of the trial towards its overall objectives, review accrual and results of the trial, adherence to the protocol, and consider any new information of relevance to the trial and the research question The TSC has advisory responsibility for the continuation of the trial. The DMC will make recommendations to the TSC who will in turn make recommendations to the Sponsor, via the TMG. The ultimate responsibility for the conduct of the trial, however, rests with the Sponsor
Membership	Professor Maria A. Hawkins, Professor in Radiation Oncology, University College London—Chair and radiation oncology expert Marianne Grønlie Guren, Consultant in Radiation Oncology, Department of Oncology and K.G. Jebsen Colorectal Cancer Research Centre, Oslo University Hospital—Radiation oncology expert Mr Dale Vimalchandran, Consultant Colorectal Surgeon, Countess of Chester Hospital—Colorectal surgery expert Mr Matt Lee, NIHR Clinical Lecturer in General Surgery, University of Sheffield—Colorectal surgery expert Mr Chris Hurt, Senior Research Fellow in Statistics, University of Cardiff—Statistical expert Mrs Ann Russell, Cancer Patient Partnership Group, Addenbrooke's Hospital—Patient representative Mr Simon Bach—STAR‐TREC Chief Investigator, Consultant Colorectal Surgeon, Queen Elizabeth Hospital Birmingham—Sponsor representative (non‐voting member)

### Ethics and dissemination

The medical ethics committees of each participating country have approved the study protocol. The UK Research Ethics Committee (REC) approval reference is 16/EM/0186. It is the responsibility of the national coordinating centre to obtain country‐specific approval and the responsibility of the Principal Investigator to obtain local approval.

## DISCUSSION

The feasibility of organ preservation using CRT has been proven, both in the setting of locally advanced disease via the international watch and wait registry [6] and also for early‐stage disease via a number of small randomized and non‐randomized trials [9, 10, 14. For patients with locally advanced yet resectable rectal cancer (chemo)radiation is used routinely with the primary purpose of reducing the risk of pelvic relapse following TME surgery. Development of cCR in the interval prior to surgery enables a small proportion of patients to consider the option of secondary organ preservation. Registry data suggest that individuals who achieve cCR may safely defer surgery to avoid the morbidities associated with TME and stoma formation, so maintaining anorectal function and HRQoL [6]. Treatment of early rectal cancer follows a different pathway as the majority of patients receive primary TME surgery [2] and National Institute for Health and Care Excellence (NICE) guidelines state that CRT should not be employed for early‐stage rectal cancer outside of appropriate clinical trials [4], where the primary purpose is to preserve the rectum either via non‐operative management or local transanal excision. This paradigm shift in the management of early rectal cancer aims to offer TME surgery only in cases deemed at high risk of local failure following CRT or where follow up detects early stages of tumour regrowth.

To date several relatively small studies evaluating primary organ preservation for early‐stage rectal cancer have reported mixed results. Importantly, there is no evidence that primary organ preservation is oncologically unsafe [9, 10, 11, 20, but more evidence from randomized trials is undoubtedly needed, particularly for those who relapse. Studies evaluating standard CRT and intensification of CRT to achieve organ preservation have reported problematic acute toxicities that negate the principle aim of therapy, to deliver treatment that is more tolerable than TME surgery [12, 13, 14. TREC has recently shown that primary organ preservation therapy incorporating SCRT and TEM achieves high levels of compliance, low toxicity and high organ preservation rates with a minimal impact on patients' HRQoL compared to TME surgery [20].

Results of the TREC study support further evaluation of SCRT, as an alternative to traditional downstaging CRT, for primary organ preservation in patients with early‐stage rectal cancer in STAR‐TREC. STAR‐TREC also introduces new measures for all patients to further reduce the impact of organ‐preserving treatment, such as smaller mesorectal (only) radiotherapy fields specifically risk‐adapted for early tumours. Moreover, standardized response assessment up to 20 weeks is performed to improve identification of cCR and consequently increase non‐operative management. Lastly, an exploratory biomarker‐driven approach is introduced to determine the feasibility of initiating surgical intervention based upon the presence of cftDNA, thereby avoiding overtreatment of cases where no residual tumour exists. Optimization of the organ preservation rate for each treatment strategy is a key area for improvement as failure of organ preservation most frequently follows conversion to TME based upon the perceived future risk of local relapse in patients with no objective evidence of residual tumour, while salvage TME for local failure is comparatively rare [13, 14, 20.

In summary, STAR‐TREC is an international study to evaluate the effectiveness of two contrasting CRT schedules, optimized for treatment of early‐stage rectal cancer to achieve organ preservation (at 30 months). Key secondary end‐points are the rate of non‐operative management, patient‐reported treatment‐related toxicity, HRQoL and non‐regrowth pelvic tumour control to 36 months. STAR‐TREC also incorporates new exploratory biomarker work to develop objective evidence for escalation of surgical therapy. The trial will yield important information to guide routine management of patients with early‐stage rectal cancer.

### Protocol amendments

STAR‐TREC phase III was implemented via protocol version 4.0, 10 October 2019 (copy available upon request).

Protocol amendments which may impact the conduct of the study, potential benefit of the patient or may affect patient safety, including changes of study objectives, study design, patient population, sample sizes, study procedures or significant administrative aspects are discussed and agreed by the International Trial Management Group (TMG) composed of the Chief Investigator, NCC representatives (multidisciplinary), patient representative(s) and the CRCTU team (Trial Statistician, Trial Management Team Leader (or deputy), Trial Coordinator, Clinical Trials Monitor). Where required, the independent TSC members are also asked to comment and approve suggested protocol amendments. All substantial amendments are sent to and reviewed by the relevant REC and/or competent authority and trial funders for approval as required; amendments are distributed to sites and implemented following receipt of approvals. Trial registries are updated periodically to ensure that trial information is up to date.

### Consent and confidentiality

The University of Birmingham is the Data Controller for this trial. Personal data recorded on all documents will be regarded as strictly confidential and will be handled and stored in accordance with the General Data Protection Regulation (EU) 2016/679 and the UK Data Protection Act 2018 (GDPR).

## ACCESS TO DATA AND SPECIMENS

Requests for access to trial data and translational biobank specimens will be considered on a case‐by‐case basis and approved in writing where appropriate, after formal application to the TMG and TSC. Considerations for approving access are documented in the TMG and TSC terms of reference. Following conclusion of the trial, anonymized data are likely to be added to an open access repository.

## POST‐TRIAL CARE

Patients will revert to local standards of care.

## DISSEMINATION

The results of this trial will be submitted for publication in peer‐reviewed journals and presentation at relevant open meetings. Closed meetings will be held after the end of each phase of the trial to allow discussion of the main results among the collaborators prior to publication. Publications will conform to the International Committee of Medical Journal Editors guidelines. When manuscripts are submitted, the corresponding author will specify the name of the STAR‐TREC group, and clearly identify the group members who can take credit and responsibility for the work as authors. The results of the trial will also be published on a clinical trials registry, and a lay summary made available on the Cancer Research UK website and via press outlets.

## AUTHOR CONTRIBUTIONS

SPB, DSM, HdW, KLG‐S, CM, FP, AG, MT, AA, NW, VH, SG, LN‐N, IT each made substantial contributions to the design of this work, drafting and revising of the manuscript, and approval of the final published version; and agreement to be accountable for all aspects of the work in ensuring that questions related to the accuracy and integrity of any part of the work are appropriately investigated and resolved. NK contributed to drafting and revising the manuscript.

## ETHICAL APPROVAL

The authors have no relevant conflicts of interest or financial ties to disclose.

## CONFLICT OF INTERESTS

No conflicts.

## Supporting information

Appendix S1

Appendix S2

Appendix S3

Appendix S4
