# Supplementary material for: Can we Save the rectum by watchful waiting or TransAnal surgery following (chemo)Radiotherapy versus Total mesorectal excision for early REctal Cancer (STAR‐TREC)? Protocol for the international, multicentre, rolling phase II/III partially randomized patient preference trial evaluating long‐course concurrent chemoradiotherapy versus short‐course radiotherapy organ preservation approaches
Source: Colorectal Dis. 2022 Mar 24;24(5):639–51. doi: 10.1111/codi.16056 (PMC9311773; doi:10.1111/codi.16056)
Supplement: Supplementary file 2 — Appendix S2 [file CODI-24-639-s003.docx]

**STAR-TREC Collaborative group**

Writing committee

S P Bach, D Sebag-Montefiore, H deWilt, KL Garm-Spindler, C Marijnen, F Peters, P Christensen, A

Gilbert, M Teo, A Appelt, N West, V Homer, S Gates, N Christou, K Haustermans, A Wolthuis, A

Debucquoy, A Martling, E Angenete, P Lind, R Keogh, L Navarro-Nuñez

Trial Management Group

S Bach, D Sebag-Montefiore, H deWilt, KL Garm-Spindler, C Marijnen, F Peters, P Christensen, G Baatrup, I Al-Najami, I Nagtegaal, CL Andersen, I Tomlinson, A Beggs, R Fijneman, M Ligtenberg, A Gilbert, J Hill, N Fearnhead, S Korsgen, I Geh, M Teo, A Appelt, N West, P Quirke, N Abbott, A Russell, C Forde, A Pallan,

Trial Steering Committee

MA Hawkins, MG Guren, D Vimalchandran, M Lee, C Hurt, A Russell,

Data Monitoring Committee

A Mirnezami, L Hiller, AS Dhadda

UK National Coordinating Centre

Statistics

V Homer, S Gates,

Trial Management

R Keogh, L Navarro-Nuñez, S Magwaro, G Nixon, M Kaur

Programming and validation

F Shi, I Nutt, C Smith

Quality Assurance & Monitoring

S Mee, K James, J Keely

Netherlands National Coordinating Centre

H de Wilt, N Greijdanus, A de Jong

Denmark National Coordinating Centre

P Christensen, KL Garm-Spindler

Sweden National Coordinating Centre

A Martling, E Angenete, P Lind

Belgium National Coordinating Centre

K Haustermans, A Wolthuis, A Debucquoy, L Smets

Participating sites Principal Investigators, Responsible Oncologist and Responsible Surgeons

(Listed in descending order of the number of patients recruited per site, combined phase II+III)

Radboud University Nijmegen Medical Centre: Hans De Wilt, Heidi Rutten

Good Hope Hospital: Stephan Korsgen, James Good

Queen Elizabeth Hospital Birmingham: Simon Bach, Ian Geh

St James's University Hospital: Mark Teo, Julian Hance, Alex Gilbert

Leiden University Medical Center: Fabian Holman, Laura Velema

Odense University Hospital: Gunnar Baatrup, Per Pfeiffer, Issam Al-Najami

York Hospital: Dibyendu Bandyopadhyay; Nathalie Casanova

Antoni van Leeuwenhoek Hospital: Corrie Marijnen, GL Beets, Femke Peters

Colchester General Hospital: Mathew Tutton, Sadaf Usman

Aarhus University Hospital: Peter Christensen, Karen-Lise Spindler

Laurentius Hospital and Maastro Clinic: Jeroen Leijtens, Maaike Berbee

Norfolk & Norwich University Hospital: Sandeep Kapur, Debashis Biswas, Atanu Pal

Addenbrooke's Hospital: James Wheeler, Rashmi Jardon , Nicola Fearnhead

Manchester Royal Infirmary and The Christie Hospital: James Hill, Noreem Alam

Bradford Royal Infirmary: Mark Steward , Paul Hatfield

Amphia Hospital: A Ten Tije, RHPH Crolla, GP Van der schelling

Churchill Hospital: Christopher Cunningham, Clare Jacobs, Stephen Boyce

Isala Zwolle: Erik Van Westreenen, Onne Reerink

Southmead Hospital and Bristol Haematology And Oncology Centre: Kathryn McCarthy, Stephen Falk

Elisabeth-Tweesteden Ziekenhuis and Insitute Verbeete: Dareczka Wasowicz-Kemps, Robert Poorter, D Zimmerman

University Hospital of Wales and Velindre Cancer Centre: Michael Davies, Richard Adams

Diakonessenhuis and University Medical Center Utrecht: Apollo Pronk, Martijn Intven

Ninewells Hospital: Ian Sanders, Dorin Ziyaie

Vrije University Medical Centre: Jurriaan Tuynman, Debby Geijssen

Catherina Hospital: Pim Burger, Jacqueline Theuws

Medisch Centrum Leeuwarden and Radiotherapeutisch Instituut Friesland: Christiaan Hoff, Susanne Pereboom

Deventer Hospital and Radiotherapiegroep: Koen Talsma, Karin Muller

Spaarne Gasthuis: Ronald Vuylsteke

OLVG: Michael Gerhards

IJsselland Hospital and Erasmus Medical Centre: Pascal Doornebosch, Joost Nuyttens
