# Supplementary material for: Can we Save the rectum by watchful waiting or TransAnal surgery following (chemo)Radiotherapy versus Total mesorectal excision for early REctal Cancer (STAR‐TREC)? Protocol for the international, multicentre, rolling phase II/III partially randomized patient preference trial evaluating long‐course concurrent chemoradiotherapy versus short‐course radiotherapy organ preservation approaches
Source: Colorectal Dis. 2022 Mar 24;24(5):639–51. doi: 10.1111/codi.16056 (PMC9311773; doi:10.1111/codi.16056)
Supplement: Supplementary file 3 — Appendix S3 [file CODI-24-639-s001.pdf]

STAR-TREC Phase III

Statistical Analysis Plan

Can we **S**ave the rectum by watchful waiting or **T**rans**A**nal surgery following (chemo)**R**adiotherapy versus **T**otal mesorectal excision for early **RE**ctal **C**ancer?

Version:

V1.0: 7th October, 2021

**Sponsor:**	University of Birmingham
**Sponsor reference number**	RG_15-011
**CRCTU reference number**	CR3017
**Coordinating Centre**	CRCTU
**EudraCT number**	2016-000862-49

1 KEY PERSONNEL INVOLVED IN THE PREPARATION OF THE STATISTICAL ANALYSIS PLAN:

1.1 NAME	1.2 TRIAL ROLE
Victoria Homer	Trial Statistician
Prof Simon Gates	Lead Statistician
Mr Simon Bach	Chief Investigator
Leyre Navarro Nuñez	Senior Trial Coordinator

2 DOCUMENT CONTROL SHEET

2.1 STATISTICAL ANALYSIS PLAN VERSION:	2.2 REASON FOR UPDATE:
Version 0.1 21-Feb-2020	Initial draft in accordance with protocol version 4.0
Version 0.2 24-Mar-2020	Second draft after consultation with lead statistician and fixing of typos etc
Version 0.3 21-Apr-2020	Update in definitions used in the calculation of primary and secondary outcomes.<br>Inclusion of section on and adjustments to be made in light of COVID-19.
Version 0.4 28-Sept-2021	Addition of wording regarding visit window around outcomes.
Version 0.5 30-Sept-2021	Final draft after consultation with CI and in line with protocol v5.1 (03-Sept-2021)
Version 1.0 07-Oct-2021	Initial version

CONTENTS

1. Introduction 4
   1.1 Purpose of the Statistical Analysis Plan 4
   1.2 Summary of the Trial 4
      1.2.1 Trial Design 4
      1.2.2 Objectives 4
      1.2.3 Study Population 4
      1.2.4 Trial Duration 4
      1.2.5 Trial schema 5
2. Timing and Reporting of Interim and Final Analyses 6
   2.1 Interim analyses 6
   2.2 Final analyses 6
3. Recruitment and Randomisation 6
   3.1 Recruitment 6
   3.2 Randomisation 6
   3.3 Ineligible Patients 7
4. Data Quality 7
   4.1 Length of patient follow-up 8
5. Trial Population 8
   5.1 Baseline characteristics 8
   5.2 Defintion(s) of populations for analysis 9
6. Treatment Received 9
7. Toxicity and Safety Analysis 9
8. Analysis 10
   8.1 Definition and Calculation of Outcome Measures 10
      8.1.1 primary outcome measure 10
      8.1.2 secondary outcome measures 12
   8.2 Descriptive Analyses 17
   8.3 Hypothesis Testing for the Primary Outcome Measure 17
      8.3.1 Null Hypothesis 17
      8.3.2 Sample Size Determinations 17
   8.4 Hypothesis Testing for Secondary Outcome Measures 18
   8.5 Bayesian Analysis of Outcome Measures 18
   8.6 Additional Analyses 19
   8.7 Decision Criteria 19
   8.8 Subgroup Analysis 20
   8.9 COVID-19 Mitigation 20
9. Statistical Software 21
10. Storage and archiving 21
11. References 21

1. INTRODUCTION

1.1 PURPOSE OF THE STATISTICAL ANALYSIS PLAN

This Statistical Analysis Plan (SAP) provides guidelines for the analysis and presentation of results for the STAR-TREC trial. This plan, along with all other documents relating to the analysis of this trial, will be stored in the 'Statistical Documentation' section of the Trial Master File. The Trial Statistician will carry out the statistical analysis.

1.2 SUMMARY OF THE TRIAL

1.2.1 TRIAL DESIGN

International, multi-centre, open-label, rolling phase II/III trial with a partially randomised patient preference design. Patients will choose organ preservation or standard surgery. Those who prefer organ preservation will be randomised 1:1 between (i) organ preservation with mesorectal Chemoradiotherapy (CRT) versus (ii) organ preservation with mesorectal Short Course Radiotherapy (SCRT). Those who prefer standard surgery or have no preference will undergo standard Total Mesorectal Excision (TME) surgery without neoadjuvant radiotherapy treatment.

The SAP specified here is **only** pertinent to the **phase III** element.

1.2.2 OBJECTIVES

The phase III component will evaluate two contrasting organ preservation strategies (either long-course chemoradiotherapy or short-course radiotherapy) for the treatment of early stage rectal cancer in terms of organ preservation rates, toxicity (clinician and patient-reported) and Health-Related Quality of Life (HRQoL).
The phase III study will also include a standard TME radical surgery (non- randomised) comparator arm encompassing reconstructive (low anterior resection) and non-reconstructive (abdominoperineal excision, low Hartmann's procedure) approaches.

1.2.3 STUDY POPULATION

Subjects referred to either a colorectal surgeon or the colorectal cancer multidisciplinary team (MDT) with suspected early stage colorectal cancer identified (i) through the bowel screening programme, (ii) development of new bowel symptoms, or (iii) as part of a personal bowel surveillance programme.

All subjects who were recruited and randomised during the phase II element of the trial shall contribute to the patient population in the phase III here analysed.

1.2.4 TRIAL DURATION

All patients (phase II and phase III) will be followed up for 36 months from the start date of (chemo)radiotherapy or initial surgery.

1.2.5 TRIAL SCHEMA

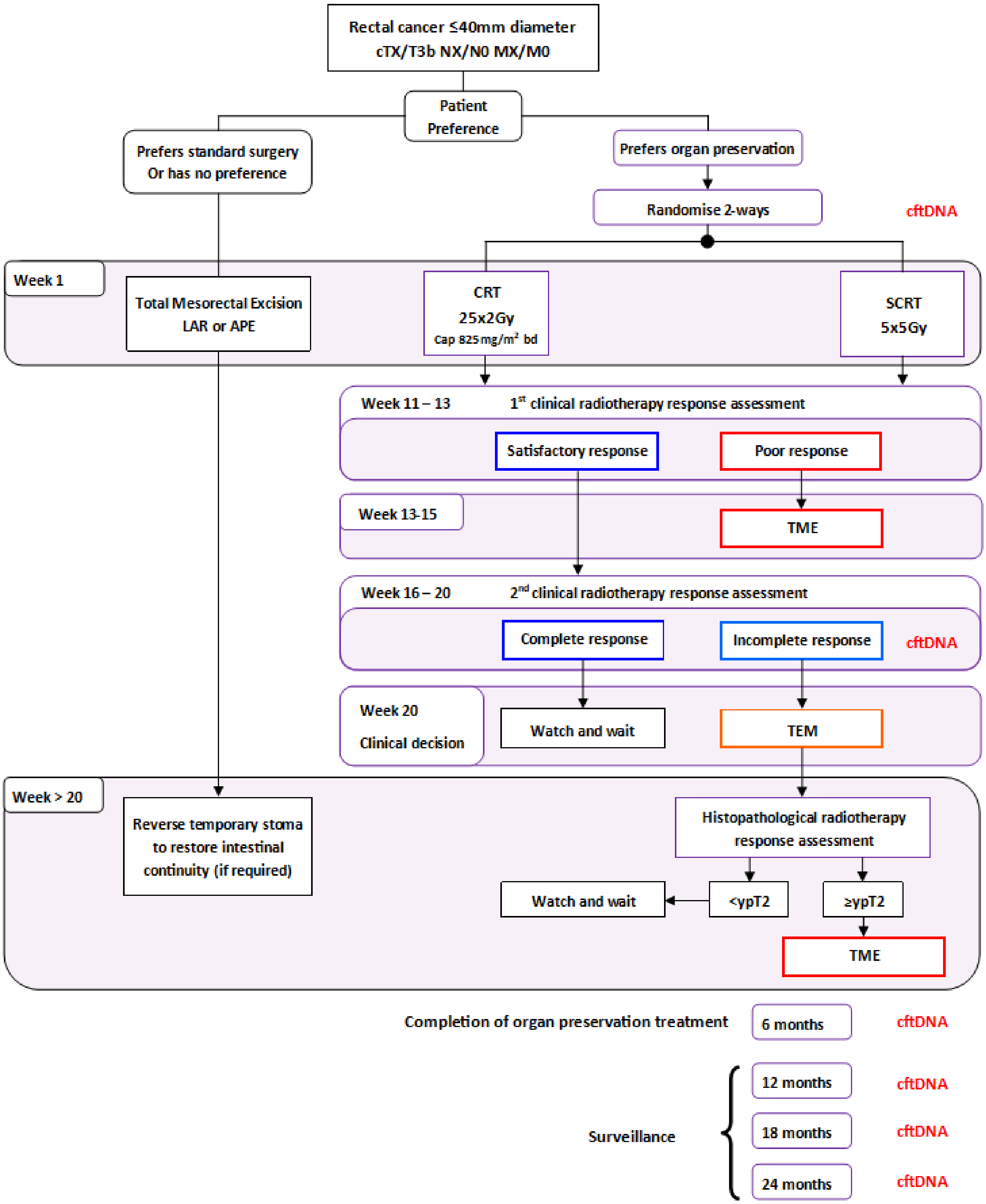

2. TIMING AND REPORTING OF INTERIM AND FINAL ANALYSES

2.1 INTERIM ANALYSES

Interim unblinded analyses of efficacy and safety will be provided in strict confidence to the independent DMC.

The DMC will meet at least annually unless there is a specific reason to amend the schedule. During the recruitment phase of the trial, the DMC is scheduled to meet six months after the recruitment of the first phase III participant and annually thereafter.

Additional meetings may be called if recruitment is much faster than anticipant and the DMC may, at their discretion, request to meet more frequently or continue to meet following completion of recruitment.

An emergency meeting may also be convened if a safety issue is identified.

The TSC for the phase III trial will meet at least once a year (usually by teleconference).

There are no formal stopping rules. The independent DMC will monitor the rates of acute toxicity, organ preservation, and pelvic relapse at regular intervals.

2.2 FINAL ANALYSES

The primary endpoint for the phase III study is 30 months from the start of (chemo) radiotherapy treatment.

The final analysis report containing all safety data, together with primary, secondary and exploratory outcomes will be prepared once all recruited patients have completed their protocol assessments.

The end of trial will be 12 months after the last data capture.

The final analysis will be reported within 12 months of the end of trial definition.

3. RECRUITMENT AND RANDOMISATION

3.1 RECRUITMENT

Typical analysis may include, but not limited to:

- Date the snapshot was taken
- Dates when the trial opened and closed for recruitment
- Recruitment over time (monthly or quarterly) and an average monthly recruitment rate
- Recruitment by site and/or clinician
- Cross-tabulation of recruitment by site / clinician (rows – in order of opening) and time interval (columns) – time when sites not open is shaded out

3.2 RANDOMISATION

The phase III element of STAR-TREC is only partially randomised. Patients will first select either:

a. Conventional TME surgery
b. Organ saving approach

Patients showing no preference will be offered to be registered to the conventional TME surgery arm.

Patients showing a preference for organ preservation will be randomised 1:1 at the time of trial entry to either:

a. Organ saving utilising chemoradiation (CRT)
b. Organ saving utilising short course radiotherapy (SCRT)

Patients randomised to CRT will receive:

- Capecitabine: 825 mg/m² orally, b.d., on radiotherapy days,
- Radiotherapy: A dose of 50 Gy applied to the primary tumour and surrounding mesorectum in 25 fractions of 2 Gy, 5 days a week.

Patients randomised to SCRT will receive:

- A dose of 25 Gy applied to the primary tumour and surrounding mesorectum in 5 fractions of 5 Gy, 5 days a week.

For patients choosing organ preservation, randomisation will be provided by a computer-generated program which will use a stratification procedure with the following variables:

1. MRI (or ERUS) Tumour staging (≤T3a / T3b)
2. Country (UK / the Netherlands / Others (currently Denmark)

Stratification will be by T stage to ensure that the more advanced tumours are equally represented across treatments. Patients that have both an MRI and ERUS performed will be stratified by the tumour stage reported from the MRI test. Stratification by country will be done to account for any bias arising from the slight differences in pre-treatment MRI based staging assessment.

To avoid any possibility of the treatment allocation becoming too predictable, a random factor will be included within the algorithm whereby for a proportion of the allocations true randomisation will be implemented rather than by using the minimisation allocation.

Analysis of randomisation will include

- Number of patients randomised by treatment group

(**Data source:** trial entry form)

3.3 INELIGIBLE PATIENTS

Ineligible patients are defined as those either registered (conventional TME surgery) or randomised (organ saving approach) patients who are subsequently found to not meet the eligibility criteria of the trial. The number of ineligible patients and reasons for their ineligibility will be reported; a sensitivity analysis may be conducted and reported if the number of ineligible patients is substantial.

Protocol deviations relating to treatment will be reported as part of treatment compliance.

(**Data source:** trial entry form, discontinuation form, deviation form)

4. DATA QUALITY

Statistical data validation is detailed in the data validation plan.

Statistical data validation will be carried out prior to any and all formal analysis, and at least once annually.

4.1 LENGTH OF PATIENT FOLLOW-UP

The length of patient follow-up shall be assessed in the following ways:

- By reporting the number of patients lost to follow-up at each follow-up visit;
- By comparing follow-up across treatment groups based on a comparison of median (IQR) length of follow-up of all patients and a reverse Kaplan-Meier analysis of all patients.

Patients lost to follow-up will not be excluded from the analysis but will be censored at the appropriate date.

(**Data source:** all trial forms)

5. TRIAL POPULATION

5.1 BASELINE CHARACTERISTICS

Baseline characteristics will be presented descriptively (without statistical hypothesis testing) on demographic, clinical baseline characteristics, and trial stratification factors. These will be stratified according to treatment received where standard surgery, SCRT, and CRT are shown in one table and using appropriate graphics. Table 1 shows an example of a table template to be used in future reports. Further covariates may be added as the discretion of the trial statistician, TMG, and DMC.

Table 1. Baseline Characteristics, stratified according to treatment allocated

	Organ Saving Approaches		Conventional TME surgery	Pooled
	CRT	SCRT		
MRI Tumour Staging (n(%))				
≤ T3a				
T3b				
Country (n(%))				
UK				
The Netherlands				
Denmark				
Other				
Sex (n(%))				
Male				
Female				
Age at trial entry*				
Median (IQR)				

* Please note, as per international guidelines, only the month and year of birth is collected. Therefore, an over-estimation is assumed with the date of birth imputed as the 1st of the month.

(**Data source:** trial entry form)

5.2 DEFINTION(S) OF POPULATIONS FOR ANALYSIS

The analyses in STAR-TREC phase III will be conducted according to the intention to treat (ITT) principle, where participants are analysed in the treatment group to which they were randomised, regardless of the treatment received.

6. TREATMENT RECEIVED

For patients opting for organ preservation, the following, stratified according to randomised treatment allocated, shall be reported:

- The number of patients receiving treatment in total and by cycle,
- The number of, and reasons for, patients not starting treatment,
- The number of, and reasons for, treatment delays,
- The percentage of protocol dose received and the range of total doses received, stratified by cycle,
- The number of, and reasons for, dose reductions,
- The level of compliance, defined as the proportion of patients who received all trial treatment without dose delays, interruptions, or discontinuations,
- The time from randomisation to first treatment,
- The proportion of patients undergoing TME as part of their primary treatment (at or before the 20 week decision point),
- The proportion of patients undergoing TME during follow up,
- The proportion of patients requiring local excision, and
- The proportion of patients requiring a stoma (separate figures will be presented for temporary and permanent stomas).

For those preferring standard TME surgery, the following shall be reported:

- The number of patients receiving surgery,
- The number of, and reasons for, patients not receiving surgery,
- The time from registration to surgery.

(**Data source:** Intraoperative form, deviation form, trial entry form, chemo-radiotherapy delivery form, short-course radiotherapy form, surgical review form)

7. TOXICITY AND SAFETY ANALYSIS

Details of all AEs and SAEs (except for planned surgery, planned hospitalisation, loco-regional or distant cancer recurrences, or death due to progression of disease) will be documented and reported from the date of commencement of protocol defined treatment until 30 days after the administration of the last trial treatment.

The following will be reported, stratified according to treatment allocated:

- ECOG performance status will be summarised according to cycle,
- The toxicities per cycle will be given as a listing of number of toxicities by CTCAE v4.03 grade and number of patients,
- A line listings given of grade 3, 4 or 5 adverse events deemed at least possibly related to treatment,
- Toxicities will be tabulated by CTCAE v4.03 grade and classification,

- Duration of adverse events will be summarised,
- For each patient, worst grade observed will be tabulated both during the trial and by cycle,
- SAEs/SARs will be reported as frequency and number of patients experiencing them, together with outcome (e.g. death, resolved etc.)

(**Data source:** Short-Course Radiotherapy Toxicity Form, Chemo-Radiotherapy Toxicity Form, Surgical Review Form, SAE form)

8. ANALYSIS

8.1 DEFINITION AND CALCULATION OF OUTCOME MEASURES

8.1.1 PRIMARY OUTCOME MEASURE

The primary endpoint of the STAR-TREC phase III study is the proportion of patients with successful organ preservation at 30 months from the start day of (chemo)-radiotherapy treatment. This endpoint will **only** be assessed for patients who prefer organ preservation.

Organ preservation is defined as an in-situ rectum (includes patients subject to transanal local resection), no defunctioning stoma and an absence of active loco-regional cancer failure.

For an individual to have **failed organ preservation** at least one of the following criteria must be satisfied:

1. At the 30-month follow-up visit: Yes to status of the patient is 'radical surgery (TME)' (**data source:** follow up form, section C);
2. At the 30-month follow-up visit: there exists an instance over all prior follow-up visits whereby the answer to 'within the timeframe of this follow-up did the patient have a stoma formed' is yes **without** suitable evidence that the stoma has been reversed. Evidence for stoma reversal shall be attained by matching dates of stoma reversal with stoma formation dates. (**data source:** follow up form, section I; intraoperative form); or
3. At any follow-up visit: Yes to 'is cancer recurrence confirmed' and 'site of confirmed recurrence' is one of
   i. 'related to surgical scar or primary tumour site'
   ii. 'mesorectal lymph nodes'
   iii. 'mesorectum vascular structures
   iv. extramesorcatal nodes
   (**data source:** follow up form, section F)

Due to the nature of the collection procedure, in the instance of a missed follow-up visit, data should be captured at the next follow-up visit. While data cleaning will be performed with the intent of minimising the possibility of missing data, if there is missing information in the definition of failure to preserve the organ at the 30 month follow-up visit, or if all information for the 30 month follow-up visit is missed, we intend to use data from CT and/or MRI scans performed up to 6 months after the theoretical 30 months' time point (or in their absence up to 6 months before this time point) for the analysis. Further details on this can be found in section 8.9.

Those patients who are lost to follow-up will omitted from the primary analysis. If the number of patients lost to follow-up is perceived to be high, this may be revised.

Individuals who have not failed organ preservation and are not a lost to follow-up will be classified as having their **organ preserved.**

The proportion of patients with successful organ preservation will be calculated as

$$Successful\ preservation\ proportion = \frac{Individuals\ with\ organ\ preserved}{All\ evaluable\ patients}$$

Where patients are evaluable if they have successful or failed organ preservation (i.e. not non-responders).

In addition to reporting the proportion of patients with successful organ preservation for each treatment arm, Bayesian logistic regression models will be used, with adjustment for baseline covariates known to be related to outcomes (such as the stratifying covariates tumour staging and country). Future versions of this document will list covariates to be adjusted for.

Let i index patient so that $i = 1, 2, \dots, N$, where N is the total trial sample size for those choosing organ preservation. Let y_i be a Bernoulli indicator variable for organ preservation where $y_i = 1$ indicates the organ was preserved and $y_i = 0$ indicates the organ was not preserved. Let x_i be an indicator variable for randomised treatment, whereby $x_i = 0$ indicates patient i received CRT treatment and $x_i = 1$ indicates patient i received SCRT treatment. Our proposed model for organ preservation is therefore:

$$y_i = \alpha + \beta x_i + \gamma_1 TumourStaging_i + \gamma_2 Country_i + \cdots + \epsilon_i$$

Where the following is true:

- $TumourStaging_i$ is the tumour staging stratification covariate for patient i,
- $Country_i$ is the country stratification covariate for patient i,
- '…' indicates other baseline covariates deemed to be associated with,
- ϵ_i are the within-group errors (assumed to be independent for different i).

A logit link will be used in the model, therefore to transform $\alpha\ and\ \beta$ into clinically meaningful parameters, the probability of organ preservation, the following transformation will be make:

$$probability = \exp\left(\frac{\alpha}{1+\alpha}\right)$$

After transformation α will represent the probability of organ preservation in the CRT treatment arm, while accounting for baseline and stratification variables.
After transformation $\alpha + \beta$ will represent the probability of organ preservation in the SCRT treatment arm, while accounting for baseline and stratification variables.

If this model does not fit the observed data satisfactorily, alternative models will be considered. Random effects, on the intercept and the effect of x_i, may be considered and if so will be assumed to be independent of the within-group errors.

Priors used will be minimally informative used primarily to regularise the posterior distribution. The prior distributions used will have low probabilities near the extreme values and rule out impossible values, but have diffuse distributions over the range of feasible values. Where possible, these will be informed by existing evidence. Full specification of the priors to be used will be given in future versions of this document.
The model will be fit in R using packages such as brms, with a sufficiently large sample and warm up.

We will report the comparison of organ preservation in each arm with the specified minimum acceptable standard (50%) as probability that it exceeds 50%.

(**Data source:** trial entry form, follow-up form, intraoperative form)

8.1.2 SECONDARY OUTCOME MEASURES

8.1.2.1 SECONDARY OUTCOMES FOR THE RANDOMISED COMPARISON BETWEEN ORGAN-PRESERVING STRATEGIES:

The following outcomes are evaluated only for individuals who chose organ preservation and have a randomised treatment allocation. The number of patients in each group shall be summarised, as shall the number of individuals who chose organ preservation but failed to be randomised.

The following endpoints shall be evaluated after all patients have received trial treatment and follow-up has been completed.

Clinician-reported acute treatment related toxicity up to 30 days following completion of (chemo)-radiotherapy

In addition to that reported in section 7, the number of dose modifications attributable to acute toxicity will be reported.

(**Data source:** Chemo-radiotherapy delivery form, Short-course radiotherapy delivery form, SAE form)

Proportion of patients with CR to (chemo)-radiation therapy

The proportion of patients with complete response (CR) shall be calculated as:

$$CR\ proportion = \frac{Complete\ response}{All\ evaluable\ patients}$$

This end-point shall be calculated at 16-20 weeks post commencement of trial treatment.
Patients who have information pertinent to this outcome missing shall be treated as non-evaluable and excluded from the analysis.
The proportion of patients with complete response shall be summarised by randomised organ-preservation strategy. Bayesian logistic regression models, with adjustment for baseline covariates known to be related to outcomes akin to the methods used in the primary outcome, will be used to assess if there is a superior treatment for proportion of patients with CR. Future versions of this document will give details on both the baseline covariates adjusted for and prior distributions.
(**Data source:** trial entry form, clinical evaluation of (chemo)-radiotherapy response form 16-20 weeks: section D)

Proportion of patients undergoing transanal local excision

The proportion of patients undergoing local excision shall be calculated as

$$Local\ excision\ proportion = \frac{Local\ excision}{All\ evaluable\ patients}$$

The number of patients who have local excision shall be cross tabulated with the associated with each follow-up time point. Analogous decisions regarding non-responders shall be made as in the primary end-point. A repeated measures plot may be produced showing the number of patients undergoing local excision at each follow-up time point.

Patients who have information pertinent to this outcome missing shall be treated as non-responders and excluded from the analysis.

The proportion of patients with local excision shall be summarised by randomised organ-preservation strategy.

Bayesian logistic regression models, with adjustment for baseline covariates known to be related to outcomes akin to the methods used in the primary outcome, will be used to assess if there is a superior treatment for proportion of patients with CR. Future versions of this document will give details on both the baseline covariates adjusted for and prior distributions.

(**Data source:** trial entry form, follow up form: section C)

Time to event of organ loss assessed for patients who prefer organ preservation; defined as the length of time from the start date of trial treatment until TME surgery

Time to event of organ loss is defined as the length of time from the start date of trial treatment until TME surgery. For patients who are randomised to SCRT, the start date of treatment is the date SCRT commenced; for patients who are randomised to CRT, the start date of treatment is the date of first radiotherapy dose this week for week 1. Where patients do not undergo TME, they will be censored at the date of their last trial related visit.

(**Data source:** trial entry form, short-course radiotherapy delivery form, chemo-radiotherapy delivery form, follow-up form)

Non-regrowth pelvic tumour control to 36 months

Non-regrowth pelvic tumour control to 36 months; defined as the length of time from the start date of trial treatment until death (any cause) or development of unequivocal pelvic recurrence but not including patients who developed local regrowth which was resected with clear margins using standard TME surgery.

The date of pelvic recurrence is taken to be the ‘date of confirmed recurrence’ (follow-up form, section F) where the disease recurrence is unequivocal. In order for disease recurrence to be unequivocal, the cancer recurrence needs to be confirmed by either endoscopy, endoluminal biopsy, CT, MRI, ERUS, radiologically guided biopsy, PET, or other confirmation method (not suspected).

For patients who are randomised to SCRT, the start date of treatment is the date SCRT commenced; for patients who are randomised to CRT, the start date of treatment is the date of first radiotherapy dose this week for week 1. Where patients do not develop unequivocal pelvic recurrence, they will be censored at the date of their last trial related visit.

Patients that developed local regrowth that is removed via standard TME surgery (“Conversion TME resection after local excision” and either Anterior resection/Abdominoperineal excision/Hartmann’s) will not be considered to have failed this outcome. Therefore, to have failed this outcome there must existence of evidence of regrowth removed via ‘salvage TME resection for tumour recurrence/regrowth’ (intraoperative form, section A) using the ‘beyond TME’ technique (intraoperative form, section B). For all such individuals, there should also exist a surgical review form whereby the ‘surgical treatment being reviewed’ is ‘salvage TME resection for tumour recurrence/regrowth’ and the appropriately timed follow-up form should indicate there has been additional surgical treatment following tumour recurrence (follow-up form section H).

(**Data source:** trial entry form, short-course radiotherapy delivery form, chemo-radiotherapy delivery form, death form, follow-up form, intraoperative form, surgical review form)

Metastasis free survival time to 36 months

Metastasis free survival is defined as the length of time from the start of trial treatment until death from any cause or detection of distant metastasis. For patients who are randomised to SCRT, the start date of treatment is the date SCRT commenced; for patients who are randomised to CRT, the start date of treatment is the date of first radiotherapy dose this week for week 1. Detection of distant metastasis shall be identified from the pathology form and the date of metastasis shall be taken to be the date of procedure (not the date of pathological assessment).

(**Data source:** trial entry form, short-course radiotherapy delivery form, chemo-radiotherapy delivery form, death form, pathology form (section 12))

Non-regrowth-disease free survival time to 36 months

Non-regrowth is defined as the length of time from the start of trial treatment until death (any cause), detection of local pelvic recurrence or distant metastasis **but not including** patients who developed local regrowth which was resected with clear margins using standard TME surgery.

For patients who are randomised to SCRT, the start date of treatment is the date SCRT commenced; for patients who are randomised to CRT, the start date of treatment is the date of first radiotherapy dose this week for week 1.

The date of pelvic recurrence is taken to be the ‘date of confirmed recurrence’ (follow-up form, section F). Detection of distant metastasis shall be identified from the pathology form and the date of metastasis shall be taken to be the date of procedure (not the date of pathological assessment). Patients who have failed this outcome will be identified using an analogous definition as given in the definition of non-regrowth pelvic tumour free survival at 36 months.

(**Data source:** trial entry form, short-course radiotherapy delivery form, chemo-radiotherapy delivery form, death form, pathology form (section 12))

Overall survival to 60 months

Overall survival is defined as the length of time from the start date of trial treatment until death from any cause. For patients who are randomised to SCRT, the start date of treatment is the date SCRT commenced; for patients who are randomised to CRT, the start date of treatment is the date of first radiotherapy dose this week for week 1. Where there is no death record, patients will be censored at the date of their last trial related visit.

(**Data source:** trial entry form, short-course radiotherapy delivery form, chemo-radiotherapy delivery form, death form)

8.1.2.2 SECONDARY ENDPOINTS FOR ANALYSES INCORPORATING THE NON-RANDOMISED STANDARD SURGERY COMPARATOR:

The following outcomes are evaluated for all individuals and shall compare those who chose organ preservation (either randomised treatment), with those who chose standard radical surgery. The number of patients in each group shall be summarised.

It is expected that low numbers of patients will chose standard surgery (as this represents the current standard of care and the burden of being on the trial (such as increased hospital appointments and quality of life forms) may outweigh any benefits of receiving standard of care on a clinical trial). Therefore, while models may be mentioned below to compare the non-randomised standard surgery comparator, if the numbers of patients choosing standard surgery is too low, descriptive analysis will instead be used. Where models are used, we acknowledge that the presence of confounding variables will play a greater role. Therefore, discussions shall take place to identify all confounders and every effort shall be made to adjust for them. Future versions of this document will list all confounders to be adjusted for.

The following endpoints shall be evaluated after all patients have received trial treatment and follow-up has been completed.

Clinician-reported acute treatment related toxicity up to 30 days following completion of (chemo)-radiotherapy or date of initial surgery

Analogous definitions and methods to those defined in section 8.1.2.1 shall be used here.
(**Data sources:** chemo-radiotherapy delivery form, short-course radiotherapy delivery form, surgical review form, SAE form, trial entry form)

Non-regrowth pelvic tumour control to 36 months

Analogous definitions and methods to those defined in section 8.1.2.1 shall be used here.
(**Data sources:** trial entry form, short-course radiotherapy delivery form, chemo-radiotherapy delivery form, death form, follow-up form, intraoperative form, surgical review form)

Metastasis-free survival time to 36 months;

Analogous definitions and methods to those defined in section 8.1.2.1 shall be used here.
(**Data sources:** trial entry form, short-course radiotherapy delivery form, chemo-radiotherapy delivery form, death form, pathology form (section 12))

Disease-free survival time to 36 months;

Analogous definitions and methods to those defined in section 8.1.2.1 shall be used here.
(**Data source**: **:** trial entry form, short-course radiotherapy delivery form, chemo-radiotherapy delivery form, death form, pathology form (section 12)

Overall survival to 60 months

Analogous definitions and methods to those defined in section 8.1.2.1 shall be used here.
(**Data source:** trial entry form, short-course radiotherapy delivery form, chemo-radiotherapy delivery form, death form)

Decision regret at 24 months

Decision regret is assessed through a quality of life questionnaire. Therefore, answers shall be transformed according to the relevant reference document/user manual. Derived indices shall be summarised at 12 and 24 months, stratified according to allocated treatment and plotted as repeated measures over time. The derived indices may then be analysed using appropriate Bayesian hierarchical models to model each patient's trajectory through time.

(**Data source**: trial entry form, regret scale)

8.1.2.3 SECONDARY ENDPOINT FOR ANALYSES OF PATIENT-REPORTED OUTCOMES INCLUDING SYMPTOMATIC TOXICITY AND HEALTH-RELATED QUALITY OF LIFE (HRQOL)

The following HRQoL questionnaires are used:

- European Organization for Research and Treatment of Cancer (EORTC) QLQ-C30
- EORTC QLQ-CR29
- EuroQoL EQ-5D-3L
- The International Consultation on Incontinence Modular Questionnaire on Male Lower Urinary Tract Symptoms (ICIQ-MLUTS)
- The International Consultation on Incontinence Modular Questionnaire on Female Lower Urinary Tract Symptoms (ICIQ-FLUTS)
- Low Anterior Resection Syndrome (LARS) Score

A brief description of each is given in the trials protocol.

Analysis of patient-reported symptomatic toxicity and HRQoL health-related quality of life at 3, 12, 24 and 36 months compared to baseline will be conducted incorporating the following comparisons:

- Randomised comparison between organ-preserving strategies,
- Non-randomised comparison between organ preserving strategies and the standard surgery comparator.

Each quality of life scale shall be transformed according to the relevant reference document/user manual. Derived indices shall be summarised at each treatment visit and plotted as repeated measures over time. The derived indices may then be analysed using appropriate Bayesian hierarchical models to model each patient's trajectory through time.

In the first instance, the following shall be assumed:

- Quality of life dimension scores to be linearly related between questionnaires,
- Quality of life dimension scored to equal zero at the time of death,
- Quality of life dimensions to be static for alive patients between the last questionnaire and the date of censoring)

Future versions of this document shall give details on model specification, priors, and any covariates adjusted for.

(**Data source:** trial entry form, (other forms to be included once CRFs have been finalised))

8.2 DESCRIPTIVE ANALYSES

Proportion of patients with successful organ preservation (primary outcome) will be reported as a proportion n/N (where n is the total number of patients with successful organ preservation, and N is the total sample size) and percentage stratified according to randomised treatment, using the formulae given in section 8.1.1.

For specific outcome measure calculations, see section 8.1.

Patient demographic information will be presented using descriptive statistics and appropriate graphics.

For continuous outcomes that relate to baseline demographics, medians and IQRs will be reported. For continuous outcomes that relate to posterior summary indices, medians and 95% credible intervals will be reported.

For categorical and discrete outcomes, number, proportion, and percentages will be presented.

Definitions of how time to event outcomes will be calculated are given in the relevant subsections of section 8.1. Time to event estimates will be calculated using the methods of Kaplan-Meier and presented as the median survival in treatment arm. As all survival outcomes pertain to secondary outcomes, no formal testing shall be completed on these.

For all outcomes, the number of evaluable and non-evaluable patients will be given.

8.3 HYPOTHESIS TESTING FOR THE PRIMARY OUTCOME MEASURE

8.3.1 NULL HYPOTHESIS

As the analysis pertaining to this trial is carried out under a Bayesian framework, there will be no hypothesis testing. Instead the posterior probability of each of the organ preservation techniques being preferable. Full details are given in section 8.1.1.

8.3.2 SAMPLE SIZE DETERMINATIONS

We have not used a traditional significance test based sample size calculation, for several reasons. First, significance testing leads to problems in interpretation, such as inappropriate dichotomisation of results into “significant” (assumed to correspond to effective) and “non-significant.” Second, p-values are not clinically meaningful, and do not address the questions of clinical importance. Third, the traditional $p<0.05$ criterion for “significance” is arbitrary, and may be unlikely to be achieved in a comparison of two active treatments, which may both be good. A p-value criterion may therefore not be a sensible way of trying to identify whether either is better.

Instead, we plan to use Bayesian methods to compare the randomised groups, which produce results that are directly interpretable in clinically relevant terms. We will compute the posterior distribution for the treatment comparison, and calculate from this the probability that each of the treatments is superior. There is not a fixed criterion for considering one treatment superior, but decisions about use of one or other treatment will be informed by the probability of superiority as well as the analyses of other outcomes, cost and other relevant considerations. In the primary analyses, we plan to use weakly-informative priors, which will assign most of the probability to plausible treatment effects.

Bayesian methods do not require a pre-specified sample size; instead we have determined the trial size by pragmatic considerations, and by simulation of the likely effects of different total numbers of recruits. A target of 380 participants appears achievable and gives the trial a high probability of being able to identify a treatment that is

substantially superior. In this appendix we provide further information about the simulations and performance of the chosen trial design.

Simulations

The simulations were conducted using the software package FACTS version 6.2 (Berry Consultants, Austin, Texas). We simulated trials with 380 recruits and 8% dropout (expected to result in final sample size of close to 350) treatment differences of 5%, 10% and 15% (absolute) with an incidence in the lower group of between 50% and 80%. Each scenario was simulated 10000 times, and we recorded the proportion of simulations that found >70%, >80% and >90% probability of benefit to the superior treatment, and the proportion incorrectly identifying the worse treatment as superior.

		Probability of superiority					
		>70% probability		>80% probability		>90% probability	
Arm 1	Arm 2	Correct arm	Incorrect arm	Correct arm	Incorrect arm	Correct arm	Incorrect arm
50%	55%	0.6578	0.0693	0.5373	0.0366	0.3720	0.0141
50%	60%	0.9145	0.0087	0.8540	0.0033	0.7235	0.001
50%	65%	0.9883	0.0003	0.9750	0.0002	0.9414	0
60%	65%	0.6699	0.0721	0.5494	0.0381	0.3823	0.014
60%	70%	0.9224	0.0065	0.8689	0.0026	0.7523	0.0006
60%	75%	0.9924	0.0001	0.9837	0	0.9561	0
70%	75%	0.7038	0.0624	0.5861	0.0305	0.4114	0.0103
70%	80%	0.9485	0.0042	0.9075	0.0016	0.8109	0.0002
70%	85%	0.9974	0	0.9944	0	0.9834	0
80%	85%	0.7604	0.0407	0.6506	0.0198	0.4737	0.0064
80%	90%	0.9847	0.0010	0.9666	0.0003	0.9169	0
80%	95%	1	0	1	0	0.9993	0
50%	50%	-	0.6079	-	0.3981	-	0.1983
60%	60%	-	0.6049	-	0.4119	-	0.2079
70%	70%	-	0.6058	-	0.4000	-	0.2035
80%	80%	-	0.5957	-	0.3996	-	0.2023

During phase III, a total of 300 patients will be randomised internationally to the organ preservation arms. An estimate of 80 patients will be recruited internationally to the comparator standard surgery arm. Recruitment period will be 4 years.

8.4 HYPOTHESIS TESTING FOR SECONDARY OUTCOME MEASURES

No hypothesis testing will be done for secondary outcomes

8.5 BAYESIAN ANALYSIS OF OUTCOME MEASURES

All the aforementioned Bayesian analysis will be conducted using Stan through R.

If any of the above mentioned models do not fit the observed data satisfactorily, alternative models will be considered. First, alternative specifications for any fixed-effects will be considered. Analytical functions of time-varying covariates will be considered (e.g. $Time^2$, or $\sqrt{Time}$) to address the potential of non-linear progression.
Secondly, and where appropriate, alternative specifications for the random-effects will be considered. It is anticipated that the terms used in the random effects structure will be a subset of those used in the fixed effects structure.
In all situations, a saturated model is likely to provide a good fit. However, we will prefer a more efficient model with fewer parameters, if possible.
In all cases, the final functional form of the models used will be presented.

For all the Bayesian analysis listed above, sensitivity to prior distribution will be assessed.

While all models will be run on multiple chains, and a warm-up sample discarded with the aim of minimising the possibility of non-convergence, non-convergence is possible. Model convergence will be assessed through visual inspection of history, density, and autocorrelation plots. Model convergence statistics of $\hat{R}$ and the effective sample size will also be monitored. As with all convergence plots, such methodology is only appropriate for detecting non-convergence, should any of the aforementioned convergence plots or statistics suggest evidence of non-convergence, sensitivity to warm-up and sample, inclusion of different baseline covariates, and alternative model specifications will be considered.

8.6 ADDITIONAL ANALYSES

Additional exploratory analysis will look into the timing of organ loss. The 2-part modelling techniques used will model the probability of organ loss, and the timing to removal for those patients that do not achieve organ preservation.

Future versions of this document will provide more details on the planned additional and exploratory analysis.

While efforts will be made to minimise the potential of missing data (through data cleaning), if there is a significant amount of missing data pertaining to either the primary or secondary outcomes, the imputation of missing data may be considered. If this is the case, the amount of missing data, and where possible, the reasons why the data is missing will be reported.

8.7 DECISION CRITERIA

In regards to identifying a superior treatment for the primary outcome, there is no explicitly known or pre-specified criteria. From conversations with clinicians and the clinical community it would be expected that a 10-15% difference between the treatment arms would be regarded as clinically important. However, for one treatment to be defined as 'superior' in this trial we shall use a patient-centred ranked composite outcome.

The patient-centred ranked composite outcome analysis will classify each patient's overall outcome, based on mortality, organ preservation, stoma formation, treatment-related toxicity, need for surgery and quality of life, into an ordinal scale. The ranking of outcome categories will be determined by consensus among the investigators during the conduct of the trial, and will be reviewed (and potentially modified) by a sample of clinicians and patients. Thus the full description of such an outcome including methods and ranking will be specified in future versions of this document, and continually re-assessed as such conversations arise. The ordinal overall outcome measure will be used to compare the randomised groups, using ordinal regression models. The main advantages of this approach are that it is more relevant to patients because it considers patients' overall outcome, and it enables all patients' outcomes to contribute to the analysis

Therefore, 'superiority' of one organ preservation treatment will require clinical judgement both from the team involved and the wider community.

Future versions of this document shall include conclusions of such conversations, how the patient-centred ranked composite outcome will be calculated, and any analysis that will be performed on it.

8.8 SUBGROUP ANALYSIS

Subgroup analyses will be performed by

i) stratification factors (country and tumour staging); and
ii) translational ctDNA results obtained as part of the trial sub-study (samples taken at 6 time points: baseline, week 16-20, and at 6, 12, 18, and 24 months).

All subgroup analyses will only test the primary outcome of the proportion of patients with successful organ preservation at 30 months. This will only be assessed for patients who prefer organ preservation and are thus randomised to either SCRT or CRT.

Analysis carried out will be descriptive only.

While it is intended that hierarchical models shall be used for such analyses, future versions of this document shall include further details.

(**Data source:** trial entry form, follow-up form)

8.9 COVID-19 MITIGATION

With the COVID-19 outbreak of early 2020 and due to the increased risk of COVID-19 infection linked to multiple hospital visits and the immunosuppressive effects of chemotherapy, from 24th March, 2020 recruitment to the organ preservation with long-course chemoradiotherapy arm (CRT) was temporarily suspended until 27th July 2020.

Patients recruited to the trial during the suspension period were offered the organ preservation choice (a choice now between the current standard of care (TME radical surgery arm) and organ preservation). Patients choosing organ preservation were not randomised as expected as per the current protocol. Instead, they were automatically allocated the short course radiotherapy arm (SCRT). Although the TME arm would remain open, this choice was not actively encouraged due to the increased risk of post-surgical COVID-19 infection.

This suspension of randomisation only affected 1 patients, and therefore no adjustments to analysis will be made as a result. If further suspensions occur, they will be listed and details given about adjustments to analysis.

The COVID-19 pandemic significantly delayed the review and approval of the substantial amendment that implemented the Phase III design (protocol version 4.0 dated 10th October 2019) at participating sites. This protocol version added a 30 months time point to the schedule of events including patient assessments required for the analyses of several trial outcomes. As a consequence, a potential risk for certain 30 month assessments to be missed or to occur slightly before or after the specified 30 month time point was identified. In order to overcome this issue, a mitigation strategy has been implemented by which we intend to use data from CT and/or MRI scans performed up to 6 months after the theoretical 30 months time point (or in their absence up to 6 months before this time point) for the analysis of outcomes which are dependent on assessments expected at 30 months after the start of (chemo)radiotherapy. The 6 months limit has been established to ensure the scans used for the analyses remain clinically relevant.

9. STATISTICAL SOFTWARE

Analysis will be conducted in R using through RStudio and appropriate packages, Stata or SAS.

Statistical software including version number will be documented on all reports and publications.

10. STORAGE AND ARCHIVING

Snapshots of data for analyses related to DMC meetings will be stored in:

S:\Stats\Shared\Trials Work\TEAM A\Colorectal\StarTrec\StarTrecAnalysis\DMC\YYYY-MM-DD\Snapshot\YYYY-MM-DD\Data\

Snapshots of data for analyses related to the end of trial report will be stored in:

S:\Stats\Shared\Trials Work\TEAM A\Colorectal\StarTrec\StarTrecAnalysis\EndOfTrial\YYYY-MM-DD\LockedDataset\YYYY-MM-DD\Data

11. REFERENCES
