# Supplementary material for: Can we Save the rectum by watchful waiting or TransAnal surgery following (chemo)Radiotherapy versus Total mesorectal excision for early REctal Cancer (STAR‐TREC)? Protocol for the international, multicentre, rolling phase II/III partially randomized patient preference trial evaluating long‐course concurrent chemoradiotherapy versus short‐course radiotherapy organ preservation approaches
Source: Colorectal Dis. 2022 Mar 24;24(5):639–51. doi: 10.1111/codi.16056 (PMC9311773; doi:10.1111/codi.16056)
Supplement: Supplementary file 4 — Appendix S4 [file CODI-24-639-s002.pdf]

Delete this line, then print on Trust headed paper

STAR-TREC

Can we Save the rectum by watchful waiting or TransAnal surgery following (chemo)Radiotherapy versus Total mesorectal excision for early Rectal Cancer

Informed Consent Form
Version 5.0, 19-Nov-2020

Site: .... **Patient Trial Number:** | | | | |

Principal Investigator: .... **Trial Reference Number:** 2016-000862-49

Please initial each box

1. I confirm that I have read and understood the Patient Information Sheet (version .... dated..........................) for the **STAR-TREC** study. I have had the opportunity to consider the information, ask questions and have had these answered satisfactorily. ☐

2. I understand that my participation in this study is voluntary and that, if I take part, I am free to withdraw at any time, without giving a reason, and without the standard of my medical care or legal rights being affected. I understand that if I withdraw, some research may have already taken place using my samples and my data and that this research cannot be undone. ☐

3. I give permission for my name, initials, date of birth, gender, hospital number and NHS/CHI number to be given to the Trials Office when I enter the study as well as a copy of this consent form. ☐

4. I understand that relevant sections of my medical notes and/or data collected during the trial, including long-term follow up data, will be supplied in confidence to the Trials Office in Birmingham for use in the **STAR-TREC** study. This information may also be looked at by the Sponsor, regulatory authorities or NHS bodies, where it is relevant to my participation in this study. I give permission for these individuals to have access to my records. ☐

5. I understand that my GP will be informed of my participation in the study and may be contacted to provide information about my progress in confidence to the Trials Office. ☐

Original in the Investigator Site File, 1 copy in hospital notes, 1 copy to the patient, 1 copy to the STAR-TREC Trials Office

CONFIDENTIAL ON COMPLETION

6. I understand that the Trials Office may access information held by Cancer Registries, Cancer Intelligence Unit, NHS Digital, National Cancer Registration and Analysis Service (NCRAS) and other similar data sources kept by the NHS or related organisations, to keep in touch with me and to follow up on my health status.

7. I agree to provide information about my current health status and quality of life to the Trials Office by completing the trial questionnaires.

8. I agree for tissue removed for diagnostic purposes and at surgery being sent to the **STAR-TREC** central histopathology laboratory in Leeds for analysis (including the extraction, analysis and storage of my DNA). I consent for these samples, or DNA from these samples, to be used for future research projects that have obtained Research Ethics Committee approval.

9. I understand that all information and samples collected will be used for medical research only; that I will not be identified in any way in the analysis and reporting of the results; and that results from any additional research will not be recorded on my medical records.

10. I understand that anonymised data from the trial may be provided to other 3rd parties (e.g. pharmaceutical companies or other academic institutions) for research, safety monitoring or licensing purposes.

11. I accept that, in the unlikely event of loss of my capacity, the research team will retain my personal data already collected and will continue to use these data for the sole purposes for which consent was sought.

12. I agree to take part in the STAR-TREC study.

Optional

The following is optional and will not affect your entry into the trial.

Please **initial** for No or Yes in the boxes: **No** **Yes**

I agree to donate additional blood samples for research purposes. I agree for these samples to be supplied to laboratories in European institutions for analysis (including the extraction, analysis and storage of my DNA). I consent for these samples to be used for other future ethically approved research projects.

**Name of patient**	**Date**	**Signature**
**Name of person taking consent**<br>You must have signed the<br>Site Signature & Delegation Log	**Date**	**Signature**

Original in the Investigator Site File, 1 copy in hospital notes, 1 copy to the patient, 1 copy to the STAR-TREC Trials Office

CONFIDENTIAL ON COMPLETION
